# Subtle Brain Developmental Abnormalities in the Pathogenesis of Juvenile Myoclonic Epilepsy

**DOI:** 10.3389/fncel.2019.00433

**Published:** 2019-09-27

**Authors:** Maxime Gilsoul, Thierry Grisar, Antonio V. Delgado-Escueta, Laurence de Nijs, Bernard Lakaye

**Affiliations:** ^1^GIGA-Stem Cells, University of Liège, Liège, Belgium; ^2^GIGA-Neurosciences, University of Liège, Liège, Belgium; ^3^GENESS International Consortium, Department of Neurology, David Geffen School of Medicine, University of California, Los Angeles, Los Angeles, CA, United States; ^4^Epilepsy Genetics/Genomics Lab, Neurology and Research Services, VA Greater Los Angeles Healthcare System, Los Angeles, CA, United States; ^5^School for Mental Health and Neuroscience, Department of Psychiatry and Neuropsychology, Maastricht University, Maastricht, Netherlands

**Keywords:** development, juvenile myoclonic epilepsy, brain imaging, genes, physioparhology

## Abstract

Juvenile myoclonic epilepsy (JME), a lifelong disorder that starts during adolescence, is the most common of genetic generalized epilepsy syndromes. JME is characterized by awakening myoclonic jerks and myoclonic-tonic-clonic (m-t-c) grand mal convulsions. Unfortunately, one third of JME patients have drug refractory m-t-c convulsions and these recur in 70–80% who attempt to stop antiepileptic drugs (AEDs). Behavioral studies documented impulsivity, but also impairment of executive functions relying on organization and feedback, which points to prefrontal lobe dysfunction. Quantitative voxel-based morphometry (VBM) revealed abnormalities of gray matter (GM) volumes in cortical (frontal and parietal) and subcortical structures (thalamus, putamen, and hippocampus). Proton magnetic resonance spectroscopy (MRS) found evidence of dysfunction of thalamic neurons. White matter (WM) integrity was disrupted in corpus callosum and frontal WM tracts. Magnetic resonance imaging (MRI) further unveiled anomalies in both GM and WM structures that were already present at the time of seizure onset. Aberrant growth trajectories of brain development occurred during the first 2 years of JME diagnosis. Because of genetic origin, disease causing variants were sought, first by positional cloning, and most recently, by next generation sequencing. To date, only six genes harboring pathogenic variants *(GABRA1, GABRD, EFHC1, BRD2, CASR, and ICK)* with Mendelian and complex inheritance and covering a limited proportion of the world population, are considered as major susceptibility alleles for JME. Evidence on the cellular role, developmental and cell-type expression profiles of these six diverse JME genes, point to their pathogenic variants driving the first steps of brain development when cell division, expansion, axial, and tangential migration of progenitor cells (including interneuron cortical progenitors) sculpture subtle alterations in brain networks and microcircuits during development. These alterations may explain “microdysgenesis” neuropathology, impulsivity, executive dysfunctions, EEG polyspike waves, and awakening m-t-c convulsions observed in JME patients.

## Introduction

Genetic generalized epilepsies (GGEs), previously known as idiopathic generalized epilepsies, constitutes about 30% of all epilepsies. Based on the recent 2017 ILAE classification, GGEs mainly encompass four epilepsy syndromes: childhood absence epilepsy (CAE), juvenile absence epilepsy (JAE), juvenile myoclonic epilepsy (JME), and epilepsy with generalized tonic-clonic seizures (GTCS) alone. Among these GGEs, JME represents the major part (i.e., between 23 and 37%) and is lifelong with few antiepileptic drugs (AED) demonstrating good efficiency.

In this manuscript, we will review the current knowledge about JME and discuss its pathophysiology. We will start by recalling the symptoms that define the JME syndrome. Next, we will describe the cognitive and behavioral phenotypes of JME patients and their siblings. Then, we will summarize the huge literature on the morphological, molecular, and functional neuroimaging of JME. Specific attention will be given to JME genes that harbor pathogenic variants in JME patients and that demonstrate Mendelian transmission and complex inheritance. We will explain what is known about the cellular roles of JME genes and describe the consequences of the mutations. Finally, we will review the cellular correlates of structural changes observed by MRI and will advance a hypothesis on how pathogenic variants of JME genes could interfere with brain development and produce the symptoms and signs of JME.

## Description and Classification

Juvenile myoclonic epilepsy, called “Janz syndrome” in Europe and “Castels and Mendilaharsu Syndrome” in South America, was first described in 1854 by Delasiauve (“Motor petit Mal”) and soon after by Herpin in 1867 (“impulsions, commotions”) (Genton et al., 2013). JME is responsible for 4–11% of all epilepsies without specific ethnic/population group (Panayiotopoulos et al., 1994; Ganesh and Singh, 2005; Jallon and Latour, 2005; Martínez-Juárez et al., 2006; Berg and Scheffer, 2011; Camfield et al., 2013; Genton et al., 2013; Kasteleijn-Nolst Trenité et al., 2013; Asadi-Pooya et al., 2015). The age of onset is between 12 and 18 years (15 ± 3.5 years), but symptoms can occur from 6 to 36 years (Camfield et al., 2013; Kasteleijn-Nolst Trenité et al., 2013; Asadi-Pooya et al., 2015).

Based on the ILAE definition (Roger et al., 1989), and a consensus amongst epileptologists/experts, JME is characterized by myoclonic jerks (MJs) and EEG high amplitude diffuse and bilateral polyspike-waves. Both are the “conditio *sine qua non*” to define the JME syndrome. MJs, during which *full consciousness* is retained, typically occur in the half hour that follows morning awakening or after an afternoon siesta. Brief, bilateral, and arrhythmic muscular contraction with variable repetition and amplitude, predominate in the upper limbs involving, in general, the dominant hand.

In 80–95% of patients, a longer cluster of MJs occurs with increasing amplitude and frequency, until MJs lead to the tonic phase of the tonic-clonic seizure [“myoclonic-tonic-clonic (m-t-c)” grand mal in the 2017 ILAE classification]. Typical absences seizures or “petit mal” (Janz and Christian, 1957), with mild impairment of consciousness occur in 10–40% of JME cases (Genton et al., 2013; Wolf et al., 2015).

Seizures are often precipitated by lack of sleep (84%), stress (70%) or alcohol consumption (51%). Less frequently, various factors or behaviors such as hormonal shifts during menstruation, light stimulations, drug abuse, or praxis or action-programing activities trigger seizures. Response to antiepileptic drug treatment, mainly valproate, is reported as “good” but one third of JME patients are refractory and seizures recur in 80% of patients who attempt to stop antiepileptic drug treatment (Stevelink et al., 2018).

## Cognitive and Behavioral Phenotypes

Despite the fact that in its initial description, Janz and Christian (1957) observed several common personality traits such as social immaturity, hedonism or lack of ambition, and endurance, JME was considered as a benign disorder for a long time. Indeed, JME patients showed no significant differences in total intellectual quotient (IQ) score in comparison to unaffected individuals (Sonmez et al., 2004; Kim S. et al., 2007; Piazzini et al., 2008; Lin et al., 2013, 2014). However, several studies reported altered performances in neurocognitive functions and logical, visual, and verbal working memory were impacted in most cases (Sonmez et al., 2004; Pascalicchio et al., 2007; Wandschneider et al., 2013; Giorgi et al., 2016; Valente et al., 2016). Reproducible deficit in attention, but also of various executive functions such as planning, response inhibition, cognitive flexibility, and verbal fluency have been reported (Pascalicchio et al., 2007; Kim et al., 2012; Wandschneider et al., 2013; Zamarian et al., 2013; Walsh et al., 2014). Praxis induction, defined by myoclonic seizures precipitated by complex cognition-guided motor tasks, was found from 24 to 47% in JME patients (Matsuoka et al., 2000; Mayer et al., 2006; Guaranha et al., 2009).

Although not defined as a psychiatric disorder, the prevalence of psychosocial dysfunctions in JME ranges between 22 and 75% (Perini et al., 1996; de Araújo Filho et al., 2007; Camfield and Camfield, 2009; Jayalakshmi et al., 2014; Syvertsen et al., 2014; Valente et al., 2016). As reported in other epilepsies (Fiest et al., 2013), JME patients have a predominance of anxiety followed by depression, and mood disorders (de Araújo Filho et al., 2007; Jayalakshmi et al., 2014). JME patients have a tendency to often be emotionally unstable, with a greater propensity to show impulsive behavior and so, could be classified as cluster B personality (de Araújo Filho et al., 2007; Plattner et al., 2007; Moschetta et al., 2011). One study showed higher scores in all domains of impulsivity in JME compared to patients with temporal lobe epilepsy (TLE) and to controls (Rzezak et al., 2015). More recently, a study showed a higher prevalence of psychosocial difficulties (linked to impulsivity and executive dysfunction) in JME compared to other GGEs (Syvertsen et al., 2019).

Social cognitive skills have also been examined in GGE patients. A first study reported impairment in recognition of facial emotions in GGE patients whereas both facial emotion recognition and facial identity recognition were impacted in patients with mesial TLE (Gomez-Ibañez et al., 2014). Another demonstrated that emotion recognition and cognitive empathy abilities are affected (Jiang et al., 2014). Note that beside these cognitive impairments, JME patients exhibited worse performance in the Strange Story Task, and the Faux Pas Task (Giorgi et al., 2016). Realmuto et al. (2015) described impaired anger recognition but no deficit in mental state attribution task in GGE, in contrast to TLE patients that showed lower performances on both. When compared to patients with frontal lobe epilepsy and mesial TLE, GGE patients showed similar level of impairment in recognition of negative emotions.

The origin or cause of these behavioral problems in JME is still being debated. On one hand, studies have suggested that personality disorders observed in epilepsies may be genetically determined. Others suggest that such behavioral problems are the consequences of the social life and relationship difficulties produced by seizure activity (Cheung and Wirrell, 2006; Moschetta and Valente, 2013).

Some research studies evaluated the contribution of anti-epileptic drug treatment, epilepsy duration or seizure control to the behavioral problems in JME patients. Broader impairments in cognitive functions and impulsive traits were observed in JME groups with treatment refractory seizures as compared to those for whom seizure control was effective (Wandschneider et al., 2013; Valente et al., 2016). Some studies reported that dysfunctions were more frequently observed in patients with higher seizure frequency or when tests were performed close to the last seizure episode (de Araújo Filho et al., 2007; Unterberger et al., 2018). Behavioral changes evolved over the progression of the disease, as older JME subjects were more susceptible to behavioral problems, suggesting this is a dynamic process (Plattner et al., 2007; Somayajula et al., 2015).

Because JME is of genetic origin (see section “Genes Associated With JME and Their Cellular Role”) and because seizure repetitions or antiepileptic medications may contribute to the neuropsychological profile of JME patients, the study of siblings has drawn particular attention. Iqbal was the first to compare JME patients and clinically unaffected siblings to a control group during a set of neuropsychological measures. This comparison pointed out that both JME patients and siblings performed worse than controls (Iqbal et al., 2009). JME patients and siblings showed deficits during intention formation and intention execution during prospective memory assessment, with JME patients being even more affected than siblings (Wandschneider et al., 2010). A study performed on GGE cases demonstrated they were sensitive to dysfunction in verbal generation, non-verbal reasoning, attention and working memory, and that their relatives were also impacted, although less, in these tasks (Chowdhury et al., 2014). Altogether, these observations suggest JME patients and their clinically unaffected siblings present a distinct neuropsychologic profile compared to controls (Iqbal et al., 2015). These results suggest the existence of a neurocognitive endophenotype in JME i.e., a heritable trait that manifest in individuals whether or not the condition (here “being JME”) is active and that is found more frequently in non-affected family members than in the general population.

## Neuroimaging Analysis and Neuropathology

For a long time, brain of JME patients were considered “normal” from a structural point of view. However, the development of more powerful MRI machine and of computational analysis allowing quantitative measurements of brain structures revealed several small differences in JMEs brains. Since the first paper published by Swartz in 1996 (Swartz et al., 1996), a search on Pubmed for the combination of terms “brain imaging” and “JME” yielded around one hundred articles and almost all of them report subtle abnormalities in brains of JME patients. These data are summarized in the following paragraphs.

### Neuropathology

Only in extremely rare occasions do autopsy brains of JME and GGE patients become available for postmortem studies. In 1985, the first opportunities in eight GGE patients (including three JME cases) provided Meencke and Janz from the Berlin school of epileptology enough evidence to propose the concept of “slight migration disturbances” which they called “microdysgenesis” in epilepsies. They associated “diffuse increase of single dystopic neurons in the stratum molecular and in the subcortical white matter (WM) in different areas of the brain” with idiopathic or primary generalized epilepsies (Meencke, 1985). Lyon and Gastaut (1985) from the Marseilles school quickly objected to the significance of microdysgenesis in GGEs because, as they reported, the same is found in normal brains). In 1999, Meencke and Veith followed up with a similar study on 591 brains from patients with different epilepsy syndromes and 7374 control brains(Meencke and Veith, 1999). What is relevant here are brains from 12 patients with absence epilepsy and 3 brains from “impulsive petit mal” or JME, had significantly greater neuron density compared to age matched controls in stratum molecular of Broadmann areas 9, 22, and 18, in the subcortical WM of areas 10, 45, and 46 and in stratum oriens of the hippocampus (Meencke and Janz, 1984; Meencke, 1985). Berkovic and colleagues from Australia, also expressed doubt regarding the concept of “microdysgenesis,” as similar abnormalities could be seen in normal controls (Lyon and Gastaut, 1985). They also questioned the changes in neuronal densities, as this finding could not be replicated in other GGE cases (Opeskin et al., 2000).

Based on the data from Meencke and on electromyogram recordings showing that anterior horn cells of JME patients are altered suggesting a reduction of axon numbers (Altindag et al., 2007), it is possible that neurons of brains of JME patients could also be abnormal in spite of a normal MRI. Note that recently, the concept of “microdysgenesis” received more support when the ILAE classified cortical dysplasias. Type I cortical dysplasia is essentially “microdysgenesis” with cortical dyslamination and displaced neurons in the WM. Epileptologists also now recognize MRI negative cortical dysplasia which most often are type I (Blümcke et al., 2011).

In conclusion, it is important to continue neuropathological studies of JME brains so that in the future, it will be possible to confirm/refute the existence of these microscopic structural abnormalities, and if confirmed, to refine their nature (at both qualitative and quantitative level) and precise their location.

### Structural MRI Studies

#### Cortical Gray Matter

Woermann and collaborators were the first to find an increase in mesiofrontal gray matter (GM) thickening in JME patients using voxel-based morphometry (VBM). Sometimes, such thickening increase is associated with abnormal cortical organization (Woermann et al., 1999). VBM is an automated quantitative analysis used for comparison of local concentrations or volume of gray and WM in a disease group compared to controls (Duncan et al., 2016). Several subsequent studies replicated this finding of increased GM volume in the frontal cortex (Betting et al., 2006; Woo et al., 2006; Kim J.H. et al., 2007; de Araújo Filho et al., 2009; Lin et al., 2009b) whereas two failed to disclose any differences (Roebling et al., 2009; Swartz et al., 2016) and two found a reduction in the frontal cortex but also in the precentral cortex and the supplementary motor area (SMA) (Liu et al., 2011; O’Muircheartaigh et al., 2011). Using surface-based morphometry, a technique used to study cortical thickness (CT) folding and gyrification (Duncan et al., 2016), more widespread cortical thinning that involve the temporal, parietal, and occipital cortices beyond the frontal lobe was also observed (Tae et al., 2008; Vollmar et al., 2011, 2012; Kim et al., 2015; Park et al., 2016; Jiang et al., 2018). One reported multiple surface area (SA) changes and one measured cortical thickening in various brain regions (Ronan et al., 2012; Alhusaini et al., 2013). As a point of comparison, a meta-analysis of GGE brains performed by the ENIGMA-Epilepsy consortium revealed thinner precentral gyri as compared to controls (Whelan et al., 2018).

#### Subcortical Gray Matter

The most reproducible observation made on JME brains using VBM is the decreased volume of the thalamus; it has been reported in 10 studies. However, the atrophied region of the thalamus is not always the same [see (Kim, 2017) for a review]. Three studies reported volume reduction of the hippocampus (Woo et al., 2006; Lin et al., 2013; Kim et al., 2015) but one did not (Saini et al., 2013). Very interestingly, a recent study detected incomplete uni or bilateral hippocampal inversion (IHI also called hippocampal malrotation) in 50% of JME patients and their unaffected siblings (Caciagli et al., 2019). IHI is not an etiologic factor in epilepsy (Bajic et al., 2009) but is an *in utero* process (Bajic et al., 2010) which could be a sign of disturbed cerebral development leading to epilepsy (Bajic et al., 2009). Finally two investigations reported a decrease of putamen volume (Ciumas et al., 2008; Keller et al., 2011). Note that the meta-analysis cited above also reported a lower volume of the right thalamus in the GGE subgroup as compared to controls (Whelan et al., 2018).

#### White Matter

More than a dozen publications evaluated WM integrity and orientation in JME brains using diffusion tensor imaging (DTI) (Deppe et al., 2008; Keller et al., 2011; Liu et al., 2011; Vulliemoz et al., 2011; Kim et al., 2012, 2015; O’Muircheartaigh et al., 2012, 2011; Focke et al., 2014; Ekmekci et al., 2016; Gong et al., 2017; Domin et al., 2018). All of these publications reported decrease in fractional anisotropy (FA), a measure reflecting the microstructural integrity of various WM tracts. The most consistent differences were observed in the thalamo-cortical network and in the corpus callosum (i.e., the cortico-cortical network). Because the motor network is dysfunctional in JME, the structural connectivity of SMA was studied and was shown to be significantly reduced (O’Muircheartaigh et al., 2011; Vulliemoz et al., 2011). In a more detailed study, Vollmar and collaborators reported complex changes of mesial frontal connectivity. They reported a reduction of connectivity between pre-SMA and the frontopolar cortex and between the SMA and the primary motor cortex, whereas there was an increased connectivity between pre-SMA and the central region and the descendant motor pathway but also between the SMA and the temporal/occipital cortex (Vollmar et al., 2012).

Several studies also reported that the cerebellum was involved in JME. One study demonstrated a positive correlation between the cerebellar WM volume and the age of seizure onset (Park et al., 2016; Gong et al., 2017; Jiang et al., 2018). JME and GGE with GTCS each have unique anatomic substrates whereas another study could not detect differences between JME and GGE (Liu et al., 2011; Focke et al., 2014). In the future it will be of utmost interest to determine if differences of anatomical substrate between the various GGE syndromes do exist.

### Functional MRI Studies

In order to better understand the neuropsychological profile of JME patients, one study assessed working memory using a verbal and visuo-spatial fMRI paradigm. No difference on activation patterns was found between JME patients and controls (Roebling et al., 2009). With a different and probably more challenging paradigm (executive frontal lobe), it was demonstrated that activation of motor cortex, and SMA was increased in JME subjects (Vollmar et al., 2011). Vollmar et al. (2012) combined DTI with fMRI and showed that, between the prefrontal cognitive cortex and the motor cortex, there is a positive correlation between fibers density and the functional connectivity Interestingly, abnormal primary motor cortex and SMA co-activation with increased cognitive load as well as increased task-related functional connectivity between motor and prefrontal cognitive network were also observed in clinically unaffected siblings, suggesting an imaging endophenotype (Wandschneider et al., 2014). In another study, mesiotemporal function was addressed using an event-related verbal and visual memory fMRI paradigm and both patient and siblings demonstrated an atypical pattern of activation (Caciagli et al., 2019). At resting state, a reduced thalamocortical functional connectivity between anterior thalamus and medial prefrontal cortex and precuneus/posterior cingulate cortex was observed (Kim et al., 2014).

### Molecular MRI and PET Studies

Two separate studies on proton magnetic resonance spectroscopy (^1^H-MRS) detected a specific decrease of N-acetyl aspartate (NAA) in the prefrontal cortex and frontal lobe of JME patients (Savic et al., 2000; Simister et al., 2003), but the most consistent findings reported across six separate studies were a decrease of NAA level or NAA/Creatine ratio in the thalamus (Bernasconi et al., 2003; Mory et al., 2003; Helms et al., 2006; Haki et al., 2007; Lin et al., 2009a; Hattingen et al., 2014) and in the hippocampus of JME brains (Ristić et al., 2011). In addition, Glx (Glu + Gln) levels or Glx ratio have been shown to be increased in the thalamus (Helms et al., 2006), striatum and insula (Lin et al., 2009a), whereas decreased in the medial prefrontal cortex and the primary motor cortex (Lin et al., 2009a). Finally, GABA levels were reported to be decreased in the thalamus but increased in the frontal cortex (Hattingen et al., 2014). Positron emission tomography (PET) investigations using specific tracers revealed a reduction in dopamine transporter (DAT) binding sites in the midbrain, and particularly in the substantia nigra, whereas the caudate and the putamen were unaffected (Ciumas et al., 2008, 2010). A reduction in dopamine D2/D3 receptor binding sites has been demonstrated in the posterior putamen (Landvogt et al., 2010), while serotonin 1A receptor binding was decreased in the dorsolateral prefrontal cortex, the raphe nuclei, and the hippocampus of JME patients (Meschaks et al., 2005). At resting state, studies showed that ^18^Fluoro-DeoxyGlucose (^18^FDG) uptake was increased in the thalamus of JME subjects and was positively correlated with the amount of generalized spike-wave (GSW) (Kim et al., 2005). When ^18^FDG uptake was measured while a battery of executive function test was performed, the frontal lobe hypometabolic values measured in JME group could predict impairment on measures of figural fluency and cognitive flexibility (McDonald et al., 2006).

### Neuroimaging Studies of Early Onset or Drug Naïve JME Patients

The above imaging data were obtained using cross sectional studies (studies at one-time point) conducted mostly on patients suffering from recurrent seizures and/or that were under medication for many years. To decipher the origin of these differences in brain imaging and neuropsychologic findings, longitudinal experiments were designed to answer the following questions: do the brain imaging abnormalities and neuropsychologic impulsivity and executive dysfunction result from developmental variations preceding the first seizures or are they consequences of epilepsy-related factors i.e., chronic seizures and/or antiepileptic medication?

When patients were evaluated within their first year of epilepsy, a smaller thalamic volume, more frontal cerebrospinal fluid (CSF) and executive dysfunctions were already detected in JME children but not in subjects suffering from benign childhood epilepsy with centrotemporal spikes (Pulsipher et al., 2009). On the other hand, GGE cases (one third being JME) within the week of being first diagnosed, presented a reduced GM volume in the thalamus but no evidence of morphologic abnormalities in other structures such as putamen, caudate, palladium, hippocampus, prefrontal cortex, precentral cortex, or cingulate was identified (Perani et al., 2018). In new-onset JME (i.e., within 5 months after diagnosis), WM abnormalities were detected in many structures such as the prefrontal and a part of the frontal cortex, the thalamus and the corpus callosum, and these WM abnormalities were correlated with the cognitive deficits (Ekmekci et al., 2016). These results indicate that reduced GM volume in thalamus and WM abnormalities are not effects of chronic seizures or antiepileptic drug treatment.

Two studies concentrated on the dynamic changes over time in the same individuals in relation to JME development. In the first study, children diagnosed with JME within the past 12 months and a control group were followed for 2 years (Lin et al., 2014). Significant differences in psychomotor speed and response inhibition existed already at the beginning of the observation between both groups (i.e., the baseline). Although both improved over time, the JME group did not catch up to controls 2 years after diagnosis. Regarding brain MRI, abnormal patterns of structural development were demonstrated, characterized by an attenuation of age-related decline in cortical volumes (CV), particularly in the fronto-parieto-temporal cortex. This greater CV resulted more from CT than SA dysmaturation (Lin et al., 2014). In the second study, a network analysis of volume changes across cortical and subcortical structures was performed after 2 years of structural MRI measurement of new-onset JME under AED treatment. It revealed that compared to control, JME developed a network of highly correlated cortical regions dissociated from the subcortical structures, suggesting a development that is not organized (Garcia-Ramos et al., 2018).

## Genes Associated With JME and Their Cellular Role

The studies performed by Lennox on relatives and twins were the first to reveal the importance of genetics in epilepsies (Lennox, 1951). These first observations were subsequently confirmed by other investigations demonstrating a high concordance for GGE in monozygotic (MZ) twin pairs compared to dizygotic (DZ) ones, strongly supporting the primacy of genetic factors. Despite this evidence of a genetic component, finding genes for JME has been more challenging than initially expected. From these studies, it was concluded that JME is a diverse group of epilepsies that have both Mendelian and complex genetic inheritance with multiple genetic determinants as causes of the disease. A large genome wide association study (GWAS) reported a number of variants enriched in patients with GGE, the strongest of these being not ion channels such as *GREM1* (a member of the DAN family of bone morphogenic protein antagonist), *OR10S1* (a member of the Olfactory receptor family), *PPEF2* (serine/threonine phosphatase with an EF-hand domain), *CHD1* (a member of a family of protein containing a chromo and a helicase domain), and *PSME2* (subunit 2 of proteasome activator complex). Another more recent GWAS identified ultra-rare genetic variations in genes encoding GABA_A_ receptor subunits in GGE patients (Heinzen et al., 2012; May et al., 2018). Although 29 chromosome loci have been genetically linked to Mendelian forms of JME, to date, only six disease causing variants (*GABRA1, GABRD, EFHC1, BRD2, CASR*, and *ICK*) have been identified. All pathogenic variants with the exception of BRD2, are autosomal dominant traits. These genetic variations underlying JME cover only a small proportion of the JME world population. Only *EFHC1* and *ICK* have been replicated in a sizable percentage of at least two separate countries of specific ancestral origin. We review their roles in the order of their discovery.

### GABRA1

In 2002, Cosette with Guy Rouleau, reported a missense mutation (A322D) in the alpha 1 subunit of the GABA_A_ receptor *GABRA1* gene in one large French-Canadian family from Quebec. It was the first mutation to be identified in autosomal dominant JME (Cossette et al., 2002). Since then, rare coding variants GABA_A_ receptor subtypes including *GABRA1* have been associated with both benign and severe epileptic syndromes, in unspecified epilepsy and in GGE, although they remain very rare in terms of frequency (Johannesen et al., 2016; May et al., 2018).

In heterologous cells, the A322D mutation causes GABRA1 subunit misfolding and rapid proteasomal degradation (Gallagher et al., 2007). In HEK cells, the mutation reduced surface expression of the other subunits and altered cell surface composition of GABA_A_ receptors (Ding et al., 2010). Expression of this subunit is relatively low at birth but increases with development/aging and replaces GABRA2 and GABRA3 (Bosman et al., 2005). Heterozygous *Gabra1*-KO mice displayed spontaneous ethosuximide-sensitive spike and waves discharges suggesting this is an absence epilepsy gene that evolved to JME (Arain et al., 2012). The effect of the A322D mutation was also evaluated in mice. Both Het-*Gabra1*-KO and Het-*Gabra1*-AD mice displayed spontaneous absences and polyspike discharges already at P35 that persist at P120. Also GABRA1 expression level was, as expected, lower at P35 compared to WT mice, there was surprisingly no more difference at P120 (Arain et al., 2015). Recently, a *gabra1^–/–^* zebrafish mutant line was generated (Samarut et al., 2018). Although homozygotes embryos developed normally, almost all died prematurely. The *gabra1^–/–^*larvae were hypoactive and light stimulation of juvenile zebrafish induced intense seizures (Samarut et al., 2018). Brain transcriptome analysis revealed that expressions of many genes implicated in development, neurogenesis and synapse function were changed. Interestingly, GABAergic projections were less arborized, supporting axon guidance alteration. In addition GABAergic presynaptic signaling was decreased (Samarut et al., 2018). This phenotype is different to what is reported in *Gabra1*-KO mice as in the latter, synapses and GABA_A_ receptors numbers were normal probably because of a compensatory mechanism by the other subunits (Bosman et al., 2005).

### GABRD

The gene encoding the delta subunit of the GABAA receptor *(GABRD)* was initially identified as a susceptibility allele for both JME and genetic epilepsy with febrile seizure plus (GEFS+). Two different missense mutations were reported, one in the GEFS + family (E177A) and another (R220H) in the JME patients (Dibbens et al., 2004). No further replications of *GABRD* mutations have been reported in JME.

Both (E177A and R220H) mutations decreased current amplitude when expressed along with GABRA1 and GABRAB2 subunits in HEK cells (Dibbens et al., 2004). When they were expressed along with GABRA4 and GABRB2, the R220H mutation decreases both cell surface expression and mean open duration (Feng et al., 2006). *Gabrd*-KO mice has been shown to be more prone to seizures induced by pentylentetrazol (PTZ) and in the dentate gyrus, faster decay of evoked IPSPs was recorded (Spigelman et al., 2002).

### EFHC1

Several heterozygous missense mutations in *EFHC1* (EF-Hand containing-1) gene in chromosome 6p12 co-segregate in 21 affected members of six unrelated families from Belize and Mexico (Suzuki et al., 2004). Later on, new heterozygous mutations in *EFHC1* were described in other Mexican, Honduras, and Japanese families but also in 3 unrelated Italian families and in 5 patients from Austria (Stogmann et al., 2006; Annesi et al., 2007; Medina et al., 2008; Jara-Prado et al., 2012). Recently two studies performed in India described 5 missense mutations in six independent families and 13 mutations in 28 patients (Raju et al., 2017; Thounaojam et al., 2017). While pathogenic variants of EFHC1 are present in 3% of Japanese JME patients, in 7 to 9% in JME patients from Mexico and Honduras and in 5% of JME patients from India, pathogenic variants of EFHC1 may be extremely rare in JME patients from Germany while possibly absent in the Dutch, Swedish, and United Kingdom populations. A rare variant in *EFHC1* was also observed in a case of Sudden Unexpected Death in Epilepsy (Coll et al., 2016).

*EFHC1* encodes a protein containing three DM10 domains and one putative EF-hand motif but its cellular function is not yet clearly established. This protein is highly expressed in cells with motile cilia and it is found along the axoneme and at the basal body (Ikeda et al., 2003; Kilburn et al., 2007). The N-terminal region but not the DM10 domains is important for protein localization to the centrosome and axonemes (de Nijs et al., 2006; Zhao et al., 2016). Although its role in motile cilia remains unknown, its absence affects their beating frequency (Suzuki et al., 2009; Stoddard et al., 2018). On the other hand, both the human and the drosophila ortholog of EFHC1 interact directly with tubulin and associate with the mitotic spindles. Moreover, either the overexpression of pathological mutants or of truncated forms, or the knockdown using RNAi, all produced abnormal mitotic spindles in HEK cells (de Nijs et al., 2009; Rossetto et al., 2011; de nijs et al., 2012; Raju et al., 2017).

In *Drosophila*, the null allele of Defhc1.1 showed an overgrowth of the dendritic arbor of type IV neurons and an increased number of satellite bouton at the neuromuscular junction resulting in increased spontaneous neurotransmitter release (Rossetto et al., 2011). Interestingly, these phenotypes were inversed or corrected when Defhc1.1 is overexpressed in the CNS and vinblastine corrected the satellite bouton phenotype (Rossetto et al., 2011). In *Xenopus laevis*, EFHC1b morphans embryo displayed CNS and neural crest patterning defects producing noticeable effects on their morphology (Zhao et al., 2016). In mice, *in utero electroporation* (IUE) experiments demonstrated that both shRNA and overexpression of pathological mutants, but not variants, impaired radial and tangential migration of neuroblasts when analysis is performed after 3 days at E17.5 (de Nijs et al., 2009; de nijs et al., 2012). However, at P5 these neurons reached their cortical layers suggesting that migration is delayed but not blocked. Interestingly, the dendritic arbor of these neurons was less developed and their axonal growth was delayed, suggesting that later developmental step are now also affected (Wolkoff, 2014). *Efhc1*-KO mice are viable with no gross brain defect except postnatal hydrocephaly. At adult stages, both *Efhc1*-Het and *Efhc1*-KO mice developed spontaneous myoclonus and both genotypes have a greater sensitivity to PTZ induced seizures (Suzuki et al., 2009).

### BRD2

A susceptibility locus that mapped to chromosomal region 6p21 was identified as a single nucleotide polymorphism (SNP) in the promoter region of the *Brd2* (Bromodomain containing 2) gene (Pal et al., 2003). This SNP which does not segregate with affected family members, suggesting complex inheritance, is located in a CpG island and was shown to affect the methylation status of the promoter and thus, probably the expression of the gene (Pathak et al., 2018).

BRD2, also known as RING3, belongs to the BET (Bromodomains and extra-terminal domain) family of proteins. It is a transcriptional regulator that associates with transcription complexes and also acetylated histone H4 (Jones et al., 1997). It is particularly expressed in the forebrain, midbrain, and hindbrain at E9.5 (Shang et al., 2009). Null mutants are embryonic lethal and exhibited smaller size and defect closure of the neural tube (Tsume et al., 2012). Heterozygous mice were viable and did not show gross abnormalities. However, a significant decrease in the number of GABAergic neurons was reported in the neocortex, striatum, thalamus and substantia nigra reticulata (SNR). Interestingly, more than half of *Brd2*-Het female mice exhibited spontaneous seizure (Velíšek et al., 2011). Under flurothyl, decreased clonic seizure threshold was observed in males whereas tonic-clonic seizure threshold was decreased in females (Velíšek et al., 2011).

### CASR

Various missense mutations in *CASR* (Calcium sensing receptor) gene co-segregates in affected members of a three-generation family and in 5 singleton individuals from south India (Kapoor et al., 2008).

CASR is calcium–sensitive G-protein coupled receptor. One of the five mutations described in epileptic patients is located in an arginine rich motif of the c-terminus. It increased the plasma membrane abundance of the receptor and by this way, strengthen the activation of the intracellular signaling pathways (Stepanchick et al., 2010). CaSR has been proposed to modulate neurite extension and branching of both sympathetic and hippocampal pyramidal neurons (Vizard et al., 2008).

### ICK

*ICK* (Intestinal Cell Kinase) was the latest gene identified by linkage and whole exome sequencing with heterozygous pathogenic variants co-segregating with affected members of two large and 8 medium sized JME families from Mexico, Honduras, Belize, and Japan, indicating transmission of an autosomal dominant gene. A total of 21 pathogenic heterozygous missense mutations in 22 patients belonging to a cohort of 310 JME probands (7%) were reported (Bailey et al., 2018). When p.R272Q *ICK* variant is transmitted as a homozygous trait, the neonatal lethal syndrome of endocrine-cerebro-osteodysplasia (ECO) result (Lahiry et al., 2009).

ICK is a Ser/Thr-kinase that belongs to the CMGC family (Manning et al., 2002). The catalytic domain is located within the first half of the protein followed by a nuclear localization signal. CCRK kinase phosphorylates, whereas PP5 dephosphorylates, a threonine residue essential for the kinase activity (Fu et al., 2006). Three ICK substrates have been identified so far, these are Scythe, an anti-apoptotic protein, Raptor, a subunit of the mTORC1 complex, and Kif3a, a molecular motor implicated in ciliary transport (Fu et al., 2006; Wu et al., 2012; Chaya et al., 2014). ICK is localized in primary cilia and regulates Sonic Hedgehog (Shh) signaling (Yang et al., 2013; Chaya et al., 2014; Moon et al., 2014). Importantly in mice, IUE experiments demonstrated that the overexpression of pathological mutants of ICK, from both JME and ECO syndrome, impaired the division of cortical progenitors of the dorsal telencephalon, and therefore impaired the radial migration of neuroblasts (Bailey et al., 2018). *Ick*-KO mice died around birth probably because of respiratory failure but brain malformations were observed around E17.5 and ciliary defects were detected in the neural tube (Chaya et al., 2014). No gross brain abnormalities were reported for *Ick*-Het mice but their sensitivity to tonic-clonic seizures induced by isoflurane was increased (Bailey et al., 2018).

## Pathophysiology of JME

Although several pathogenic variants belonging to six different genes have been identified in JME patients, the pathophysiology of this epileptic syndrome is still unknown. The main challenge is to reconcile neuropsychologic and brain-imaging data with defects in specific steps of brain development. These specific steps also have to be reconciled with events that are taking place during adolescence as JME symptomatology starts mainly around this period of time. In this section, we will try to characterize the molecular and cellular defects that may be produced by the pathogenic variants of JME genes during the whole process of brain development in order to highlight the idea that JME is a subtle neurodevelopmental disease that occur in otherwise intellectually normal persons. JME is not simply a “channelopathy.”

### Mechanisms Involved in GM Changes

To help understand and explain the changes in GM volume observed in brains of JME patients, one has to look at the estimated cellular composition of the human neocortex. Estimation reported that neuron soma account for 8% of the GM volume, astrocyte soma for 6%, oligodendrocyte (OL) for 6%, synapses for 6%, dendrites for 30% and non-myelinated axons for 29% (Bennett, 2011). Of course it is possible that these estimates may not be strictly extrapolated to other structures such as the thalamus for example, as the ratio of glial cells vs. neurons is very different in the human cortex compared to the subcortical region (ratio of 4 and 11, respectively) (Azevedo et al., 2009). Based on these values, any processes affecting dendritic or axonal outgrowth will most probably have the strongest impact on GM volume/thickness. An early postnatal lesion of the medial dorsal thalamus leads to loss of dendrites and spines in the adult prefrontal cortex (PFC). Importantly, during early adulthood in mice, the density of amygdalo-cortical fiber increases the number of axospinous and axodendritic synapses in the neuropil (Cunningham et al., 2002; Marmolejo et al., 2013) demonstrating the complex influence of different brain regions on each other’s development. Other cell types may also contribute (probably indirectly) to these variations. For example, chronic treatment of Macaque monkeys with antipsychotic medication reduced GM volume of the left parietal lobe because of a decreased number of astrocytes but not neurons (Konopaske et al., 2008). Processes such as pruning, apoptosis and myelination of intracortical axon also contribute to GM variations.

### Mechanisms Involved in WM Changes

White matter changes observed in normal brains during adolescence are attributed mainly to the myelination process, to an increase in axon caliber as well as to the proliferation of oligodendrocyte precursors cells (OPCs). In the human brain, OPC represent 10–15% of glial cells (Staugaitis and Trapp, 2009). In the mouse brain, more than 20% of myelinating OLs are adult born (Rivers et al., 2008). The differentiation of OPC into OLs is regulated by intrinsic and extrinsic factors such as neuronal activity (Hill et al., 2014). It is accepted that OLs initially wrap processes around many axons. However, axon diameter is a core determinant of myelination in the CNS, and so, only the most active ones will trigger *de novo* myelination. A fundamental emerging concept is that OLs do provide metabolic support to the axon they myelinate as, via their NMDA receptors, they sense axon energy demand (Saab et al., 2016).

In magnetic resonance imaging DTI analysis, the FA “eigenvector” is a parameter sensitive to several tissue characteristics such as myelination status, axon diameter, fiber density, and fiber organization. These means WM components beyond myelinated axons such as astrocytes, OPC, microglia, and vasculature play a role (Walhovd et al., 2014). As discussed by Walhovd et al. (2014), in a volume of WM equivalent to one voxel in high resolution MRI of rodent brain, the number of axons varies greatly from 1000 to 13,000. There are also four times more OLs than astrocyte and almost eight times more than OPC and microglia (Walhovd et al., 2014). However, the processes of astrocytes cover a volume as large as the one covered by myelin, and so they make a significantly contribution to WM signal (Walhovd et al., 2014). Because human astrocytes are even larger than mouse astrocytes, they may have an even larger impact on WM signal if their number remains the same as in mouse (Walhovd et al., 2014). So, it will be difficult to interpret a modification in FA without concomitant information about glial cells population. In cat and monkey, it has been demonstrated that the corpus callosum undergoes a reduction of cross sectional area because of an axonal elimination that precedes the myelination process (LaMantia and Rakic, 1990). In mice, early stages of motor-skill learning require the rapid production of new OLs (Xiao et al., 2016) and low frequency brain stimulation results in an activity-dependent remodeling of myelin including OLs proliferation and an increase in myelin thickness and a decrease in axon caliber (Piscopo et al., 2018). However, in humans, the same studies cannot be performed and so, cross-sectional WM variations seen *in vivo* may not be directly related to the sole changes in number of axons or of their myelination. Progress is being made to fully characterize WM non-invasively, giving the hope that soon, we will be able to perform “*in vivo* histology of the human brain” (Campbell et al., 2018).

### Brain Development During Adolescence

It is now well established that some developmental timing in the human brain may differ significantly from other species. Notably, interneurogenesis seems to be protracted in humans (Wang et al., 2011) and probably continue at low level during adolescence. Indeed in the prefrontal cortex, the expression of calbindin and parvalbumin inhibitory neurons markers continue to increase until childhood or adolescence (Fung et al., 2010). Myelination is also developmentally protracted, even when compared to one close living phylogenetic relative such as the chimpanzee, and its time course is highly region dependant and takes place at variable rate both across and within functional circuits (Miller et al., 2012).

Interestingly, a region-dependent fraction of parvalbumin interneurons from both human and mouse brain, has been reported recently to exhibit axonal myelination with a bias toward proximal segments, a process that facilitates the propagation of action potential (Stedehouder et al., 2017). Moreover, in mice, OPC cells and their nearest interneurons form a transient structured synaptic network showing a local spatial arrangement but the effect of GABA on OPC proliferation and differentiation remains controversial (Orduz et al., 2015; Balia et al., 2017; Hamilton et al., 2017). These data suggest a more intricate relation between the GABAergic system and OLs which could have potential physiological relevance in some diseases like JME.

### Roles of Mendelian JME Genes During Brain Development

Based on the drastic phenotype of KO mice, it is clear that BRD2, and ICK proteins are both important during the first steps of brain development, when cell division, and expansion of progenitors cells are prominent. Interestingly, both heterozygous mice are viable and develop well until adulthood, indicating that developmental defects are more subtle in these heterozygous animals.

Indeed, in *Brd2*-Het mice, the number of interneurons is slightly decreased and only in some brain regions, producing a subtle change that suggest that division of interneuron cortical progenitors is impacted by *Brd2*. Division of other progenitor types may also be affected; for example, Brd2 is well expressed in OPC and OLs, and specific inhibitors of BET protein family blocks OPC differentiation into myelin-producing OLs (Gacias et al., 2014). As a transcriptional regulator, BRD2 most probably influences other cellular processes important for brain development. Their characterization will require the identification of the protein/substrate and chromatin interactions as has been done for *BRD1*, a susceptibility gene for schizophrenia and bipolar disorder (Fryland et al., 2016).

*ICK* is a kinase that modulates Shh signaling. Shh is important for neurogenesis of both projection neurons and interneurons but also for astrogliogenesis (Xu et al., 2010; Araújo et al., 2014). Moreover, *ICK* is expressed well in OLs and may affect their production and physiology in the postnatal brain (Tong et al., 2015; Sanchez and Armstrong, 2018). *ICK* also activates mTORC1, a master regulator that integrates extracellular signals with downstream processes such as cell proliferation, growth, survival, fate decision and protein synthesis (Laplante and Sabatini, 2012). Mutations in genes encoding regulatory proteins of the mTOR pathway were reported in focal cortical dysplasia (FCD) that are developmental malformations of the cerebral cortex (Iffland and Crino, 2017). In the Tsc2 ± mice, mTORC1-autophagy signaling is disturbed. This causes synaptic pruning defects, increased dendritic spine density in cortex layer V and produced autistic-like social behaviors (Tang et al., 2014).

*EFHC1* influences microtubules (MT) and their dynamics in different cell types with putative important consequences for various processes taking place during brain development such as cell division and migration of progenitors, neurite, and synaptic boutons formation. Beside its importance for division of progenitors, MT reorganization driven notably by a severing mechanism is essential for interneuron precursor migration, for dendrite/axons formation and also neuronal connectivity changes due to synapse rewiring as demonstrated in *C. elegans* (Sakakibara et al., 2013; Eom et al., 2014; Kurup et al., 2015).

The *GABRA1* and *GABRD* subunits have of course profound influence on the properties of the GABA_A_ receptor subunits that are expressed by mature neurons and so they impact neuronal excitability in response to GABA. They may also contribute to some processes important for formation of brain circuitry. Indeed, *GABRA1* and *GABRD* expression also increase gradually during postnatal ages, when migrating interneurons enter the cortex at E14.5, their sensitivity to GABA increases and this parallels the increased level of several GABA_A_ receptor subunits including GABRA1 (Cuzon Carlson and Yeh, 2011). *GABRD* could be important during adolescence. It is part of the extrasynaptic GABA_A_ receptor type that emerges on dendritic spine of CA1 during female puberty and that indirectly triggers adolescent pruning via an inhibition of NMDAR (Afroz et al., 2016).

Calcium is also of major importance and critical for brain growth and maturation. Calcium regulates gene expression and participates in dendrite development and synaptogenesis. Indeed, *CaSR* modulates neurite extension and branching in both PNS and CNS. *CaSR* regulates neuronal excitability and synaptic transmission by controlling the Na + -leak channel non-selective (NALCN). Presynaptic *CaSR* blocks the non-selective cation channel (NSCC) (Ruat and Traiffort, 2013). *CaSR* is considered as a marker of newly formed OLs (Marques et al., 2016) and affects myelin formation (Ruat and Traiffort, 2013). Finally, the absence of *CaSR* impairs neural stem cells differentiation (Liu et al., 2013).

Based on the cellular roles of proteins encoded by genes associated with JME, we hypothesize that, rather than pointing toward a single common mechanism, different and diverse but not necessarily overlapping developmental steps may be affected and may lead to, or favor, adolescent onset JME. However, much more work is needed to precisely identify the cellular and molecular details of the brain defects that are induced *in vivo*.

## Conclusion

To summarize the above data: (1) Frontal lobe dysfunction, as evidence by impulsivity and dysexecutive cognitive traits, is present in JME patients and their clinically unaffected siblings; (2) MRI and PET investigations show morphological and functional differences in patients with JME compared to controls; (3) The thalamus, frontal cortex, SMA, corpus callosum and thalamocortical tracts are the most commonly impacted structures with varying changes reported in different JME patients; (4) Structural differences are already detected when seizures start during adolescence, suggesting these structural changes precede the symptomatology of JME; (5) Five Mendelian genes (GABRA1, GABRD, EFHC1, CasR, and ICK) and one complex inheritance gene (BRD2) that statistically associate with JME more than by chance, fulfill various roles in cellular function. Some of these JME genes are expressed not only by neurons but also by OLs.

One hypothesis is that, at least, some of the etiological events that lead to JME, occur during the first steps of brain development, setting up the networks and microcircuits that drive the JME psychosocial, neuropsychological and seizure phenotypes. The effects of these early steps in brain development remain latent and are only expressed later as symptoms in the context of adolescent brain maturation and adolescent onset of JME symptomatology. The advent of higher-resolution brain imaging techniques (e.g., 7 Tesla MRI) might reveal a “better picture” of the morphological and functional abnormalities that are present in JME. In recent years, for example, we have come to realize many cases of surgically resected focal epilepsy result from MRI negative microscopic FCD. These type I cortical dysplasia can be undetectable by current 3 Tesla MRI inspection but are found by histopathological analysis, to be very similar to what was originally described as “microdysgenesis.” In autism, a similar experience has been reported. Cortical neuropathology on *post-mortem* brains revealed different cytoarchitectural defects such as alteration of neuronal densities, heterotopias or poorly defined neuronal layers but these are not detected by present brain imaging methods (Ecker et al., 2017).

In conclusion, based on present evidentiary data, JME does not result from the simplistic concept of an endogenous imbalance between excitation and inhibition thresholds of a neuron or a group of neuron(s) in an otherwise histologically normal brain. Rather, evidence points to alterations in the first steps of brain development when cell division, expansion and migration of progenitor cells, including interneuron cortical progenitors, driven by pathogenic variants of diverse JME genes, sculpture subtle alterations in brain networks/microcircuits structure, and dynamics that in turn subserve the psychosocial, cognitive and seizure phenotypes of JME. Now, we have to explain how these networks and microcircuits imbalances ignite into the symptomatology of explosive awakening myoclonias and grand mal m-t-c convulsions that are triggered by sleep deprivation, hormonal shifts and fatigue.

## Author Contributions

All authors listed have made a substantial, direct and intellectual contribution to the work, and approved it for publication.

## Conflict of Interest

The authors declare that the research was conducted in the absence of any commercial or financial relationships that could be construed as a potential conflict of interest.

## Abbreviations

AED: antiepileptic drugs
AS: absence seizure
CAE: childhood absence epilepsy
CSF: cerebrospinal fluid
CT: cortical thickness
CV: cortical volume
DTI: diffusion tensor imaging
FA: fractional anisotropy
FCD: focal cortical dysplasia
fMRI: functional magnetic resonance imaging
GEFS+: genetic epilepsy with febrile seizure plus
GGE: genetic generalized epilepsy
GGE: idiopathic generalized epilepsy
Gln: glutamine
Glu: glutamic acid
GM: gray matter
GSW: generalized spike wave
GTCS: generalized tonic-clonic seizure
IHI: incomplete hippocampal inversion
IQ: intellectual quotient
JAE: juvenile absence epilepsy
JME: juvenile myoclonic epilepsy
MBP: myelin basic protein
MJ: myoclonic jerk
MRI: magnetic resonance imaging
MRS: magnetic resonance spectroscopy
m-t -c: myoclonic-tonic-clonic
OLs: oligodendrocytes
OPC: oligodendrocyte precursor cell
PFC: prefontal cortex
PTZ: pentylenetrazol
SA: surface area
Shh: sonic hedgehog
SMA: supplementary motor area
TLE: temporal lobe epilepsy
WM: white matter.
